# Intentional Sedation as a Means to Ease Suffering: A Systematically Constructed Terminology for Sedation in Palliative Care

**DOI:** 10.1089/jpm.2021.0428

**Published:** 2022-04-20

**Authors:** Alexander Kremling, Claudia Bausewein, Carsten Klein, Eva Schildmann, Christoph Ostgathe, Kerstin Ziegler, Jan Schildmann

**Affiliations:** ^1^ Institute for History and Ethics of Medicine, Interdisciplinary Center for Health Sciences, Martin Luther University Halle-Wittenberg, Halle, Germany.; ^2^ Department of Palliative Medicine Ludwig-Maximilian-Universität, Comprehensive Cancer Centre Munich (CCCM), LMU University Hospital, Munich, Germany.; ^3^ Department of Palliative Medicine, Comprehensive Cancer Center Erlangen-EMN (CCCER-EMN), Friedrich-Alexander-Universität Erlangen-Nürnberg (FAU), Universitätsklinikum Erlangen, Erlangen, Germany.; ^4^ Department of Criminal Law, Criminal Procedural Law, Commercial Criminal Law and Medical Criminal Law, Friedrich-Alexander-Universität Erlangen-Nürnberg (FAU), Erlangen, Germany.

**Keywords:** definition, palliative sedation, sedation, terminology

## Abstract

**Background:**

Terminology concerning sedation in palliative care is heterogeneous, vague, and difficult to apply with negative impact on the reliability of quantitative data, practice, and ethical discourse.

**Design:**

To clarify the concept, we systematically developed definitions of core terms in an interdisciplinary research group comprising palliative care, ethics, law, and philosophy, integrating feedback from external experts.

**Results:**

We define terms stepwise, separating matters of terminology (What is the practice?) from matters of good practice (How to use it?). We start with an operational definition of “reduced level of consciousness” (score < 0 on the Richmond Agitation-Sedation Scale modified for palliative care inpatients (RASS-PAL), followed by defining “sedating,” “sedation,” and “intentional sedation” as the result or process of sedating a patient as a means of achieving a previously defined treatment goal and the terms “light,” “deep,” “temporary,” and “sedation until death.”

**Conclusion:**

The terminology facilitates the precise phrasing of aims, indications, and rules for good practice. Empirical research on acceptance and feasibility is needed.

## Introduction

Experts within and outside palliative care criticize the terminological situation concerning sedation in palliative care and have repeatedly requested improvement.^1,2^ Definitions differ significantly in content and structure,^3^ and there is uncertainty among practitioners about identifying cases with commonly used terms such as “palliative sedation.”^4–6^

This situation poses challenges, for example, regarding the comparison of data on the frequency of sedation in palliative care. It has a negative impact on the validity of quantitative studies and may explain inconsistent results.^7–9^ Moreover, it is also highly probable that terminological insecurity will affect the quality of care. Professionals might be hesitant to provide treatment that is indicated or might hastily apply inadequate medication—against ethical/legal restrictions in their respective context. In addition to these problems, some authors also discuss possible negative effects of the terms^2^ and definitions^10^ chosen on the ethical evaluation of sedation practices.

Although terminology for sedation in palliative care has been agreed by consensus processes,^11^ the respective definitions have, to the best of our knowledge, never been justified systematically. The aim of this study was to conduct a logically precise definition for sedation practices in palliative care.

## Methods

**FIG. 1.**
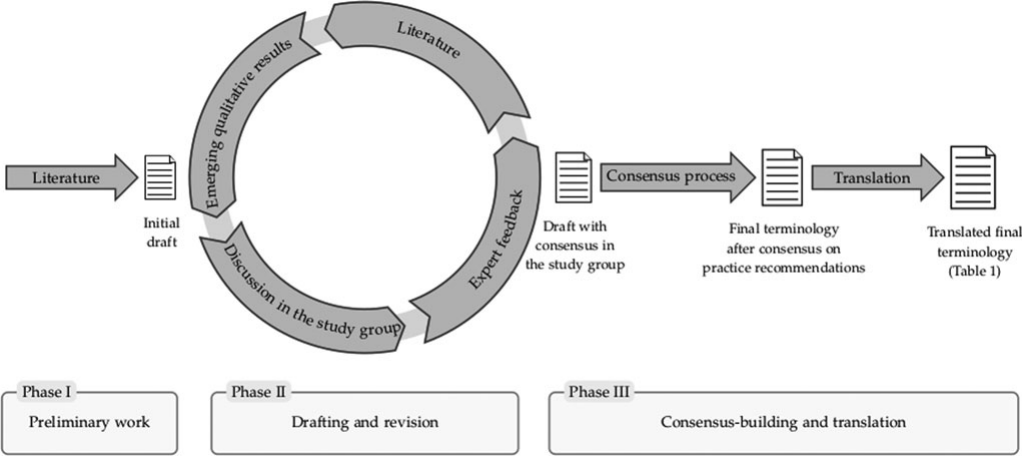
Process diagram.

There is great demand for conceptual improvements but no standardized process or reporting guideline for the development of terminology. Therefore, we based our research on a structured process of problem analyses, interdisciplinary exchange, and different strategies to secure the quality of our proposal (Fig. 1).

The study group developing the terminology comprised experts from two centers of palliative medicine, experts in medical ethics and philosophy, and experts in medical law. As a starting point, we used logical analyses of the content and structure of the definitions (or definition-like passages) identified in a systematic literature search for guidelines on sedation in palliative care.^3^ This was followed by repeated discussions within the SedPall study group, including work with case vignettes on controversial cases. We also included emerging results from the qualitative interviews on perspectives of health care professionals, relatives, and patients.

Drafts of the terminology were discussed with external experts in conferences and a workshop with external legal experts. The terminology was then presented as part of a consensus process for practice recommendations with 45 practitioners and researchers. Written and oral feedback was used to revise the terminology before it was published in the introduction of our recommendations for sedation practice.^12^ The finalized terminology was translated into English with the help of native speakers with experience in medicine or palliative care.

According to research ethics regulations, this theoretical study does not need a vote of a research ethics commission.

## Results

We decided to proceed step-by-step (constructive definition), that is, introducing terms one after another, using only expressions defined already to achieve clarity. Furthermore, we excluded matters of good practice (e.g., indication and requirements for sedation) from the core terminology. In the following, we summarize the most important implications of the terminology presented in Table 1.

**Table 1.** Terminology for Sedation in Palliative Care (Expression to be Defined—Definiendum, Defining Expressions—Definiens)

Expression to be defined	Defining expressions
Reduced consciousness	Consciousness scoring <0 on the RASS-PAL scale ( = below normal alertness)^13^
Sedated	Consciousness reduced by medical means
Sedating	Inducing a state of reduced consciousness by medical means
Sedation	Result or process of sedating
Intentional sedation	Result or process of sedating a patient as a means of achieving a previously defined treatment goal
Lightly sedated	Consciousness reduced by medical means to a score of −1 to −2 on the RASS-PAL scale
Deeply sedated	Consciousness reduced by a medical measure to a point of ≤−3 on the RASS-PAL scale
Temporarily sedated	Patient is sedated only for a certain period of time
Sedated until death	Patient is sedated continuously until his/her death

RASS-PAL, Richmond Agitation-Sedation Scale modified for palliative care inpatients.

We defined reduced consciousness by a score <0 on the Richmond Agitation-Sedation Scale modified for palliative care inpatients (RASS-PAL) scale.^13^ This implies that a patient shifting from a state of agitation to a normal level of awareness due to medication is not sedated (he/she could be called “calmed” instead). We focused on the levels <0 because limited mental and physical abilities (especially not being able to communicate and to have conscious experiences) become relevant—also for the legally crucial ability to give informed consent.

According to our terminology, sedation is the consequence of a medical intervention. Patients becoming less awake or unresponsive due to the progress of their illness are not “sedated” and not “in a state of sedation.” Thus, according to the proposed terminology proposal, sedation is a causal notion (i.e., it depends on the cause of the reduction of consciousness). We chose “medical means” because the (undisputed) fact that sedation is induced predominantly by drugs only reflects current clinical possibilities. Cases of sedation by different interventions or light sedation by hypnosis might be rare but should not be excluded by the terminology.

According to our definition, only the causal role of the clinical action is crucial for our definition of sedation. We fully acknowledge that it might be difficult in individual cases to decide whether a reduction in a patient's consciousness occurred as part of the progression of the disease or was induced by medical means. In these cases, it might even not be possible to decide whether it is a case of “sedation” or a case of reduced consciousness due to another cause.

## Discussion

Our terminological proposal is systematically and interdisciplinarily constructed. We aim at facilitating the precise phrasing of specific aims of sedation in palliative care (e.g., to reduce suffering), indications (e.g., refractory and intolerable suffering) and rules for good practice (e.g., appropriate medication). Three terminological issues warrant particular justification.

### Reasons for a terminology that is not specific for (good practice in) palliative care

Using the sedative effects of drugs is a common practice in various areas of medicine. In addition, the purpose to ease suffering by sedation is not specific to palliative care but also applies to other areas such as emergency medicine.

Instead, the unique context in which intentional sedation may take place—patients, indications, acceptable risks, and implementation, to only name a few—is specific for palliative care. Specifics for palliative care such as descriptions of patient conditions, indications, or preconditions for sedation (similar to refractoriness) are—except for the RASS-PAL score to operationalize different levels of consciousness—not part of the terminology but can be addressed and discussed in a transparent way based on the terminology.

In most guidelines, the authors have summarized parts of their understanding of clinically and ethically good sedation practice in palliative care in the respective definitions of “palliative sedation.”^3^ We deviate from this strategy for the following reasons: first, the definition of a practice should not vary between institutions/countries as a consequence of slightly different rules of good practice. Second, this strategy has caused an abundance of concurrent definitions with no tendency toward any international consensus. We believe that a descriptive definition does not exclude any normative restrictions on sedation in palliative care but instead separates normative aspects in a more comprehensive way without pre-empting ethical correctness.

### Reasons for “intentional”

By using “intentional,” we have distinguished the deliberate clinical choice of sedative effects (irrespective of the purpose) from those sedative effects that are unintended and occur during the treatment of symptoms (e.g., due to increased pain medication). There may be gray areas when health care professionals, perhaps out of pity, hope for sedative effects, when beginning a treatment aiming only at other effects (e.g., anxiolysis and pain relief). There may also be gray areas when there is, in addition, a deliberate decision to accept sedation as a side effect. The terminology triggers a decision in these cases: If a sedative effect that was initially not intended is then accepted as a means to achieve the treatment goal, then the consequence would be to proceed with the now “intentional” sedation. If the sedative effect is not accepted, then the consequence would be to take countermeasures against the unintended effect, if possible, for example, dose reduction.

We acknowledge the critical positions on the reference to intentions in definitions.^11,14^ However, we want to emphasize that the terminology is only using the legally and morally basic distinction whether an effect (sedation) was induced intentionally or unintentionally. We do not rely on specific intended treatment goals in the defining expressions of the terminology (right column of Table 1). “Intentional” has recently also been used elsewhere to characterize the practice but was not defined there.^15–17^ We offer a definition that does not contradict the fact that motivations or wishes of palliative care professionals may be psychologically multifaceted.^18^

### Reasons against “intermittent,” “continuous,” and “terminal sedation”

We avoid the term “intermittent” because it implies repeated intentional phases of sedation. Intermittent sedation might, therefore, be a special case of one or more phases of sedation and might (or might not) end in a phase of “sedation until death.” In addition, we do not use the term “continuous” because, taken literally, each sedation is “continuous” for its respective amount of time. In addition, as a term that refers to sedation until the patient dies, the term seems to be euphemistic. By contrast, our proposed term “sedated until death” describes the process as it is without implying a “termination,” in the sense of an intentional ending of the patient's life, as the term “terminal” would imply.^15^

## Conclusion

The proposed terminology is simple and consistent with other areas in medicine. It is logically precise, which is useful for operationalizing the practices of sedation in research. In addition, basic terms of the definition remain the same even when rules of good practice may differ between countries. Such neutral terminology facilitates identification and analysis of ethical and legal questions separately from the terms used to describe sedation practices. In particular, typical clinical courses with changes of treatment goals and the acceptability of sedative effects can be easily described and addressed. A limitation of the proposed terminology is the lack of empirical research on acceptance and feasibility. Accordingly, further research should address terminological reliability and applicability also in comparison with existing definitions.
